# TRIM29 overexpression is associated with poor prognosis and promotes tumor progression by activating Wnt/β-catenin pathway in cervical cancer

**DOI:** 10.18632/oncotarget.8686

**Published:** 2016-04-12

**Authors:** Rui Xu, Jingye Hu, Tiansong Zhang, Chao Jiang, Hui-Yun Wang

**Affiliations:** ^1^ State Key Laboratory of Oncology in South China, Guangzhou, China; ^2^ Collaborative Innovation Center for Cancer Medicine, Sun Yat-Sen University Cancer Center, Guangzhou, China; ^3^ Department of Basic Medicine, Guiyang College of Traditional Chinese Medicine, Guiyang, China; ^4^ Gynecology and Obstetrics Department, Women and Children's Medical Center, Guangzhou, China

**Keywords:** TRIM29, prognosis, proliferation, metastasis, cervical cancer

## Abstract

Dysregulation of TRIM29 has been reported to be involved in tumorigenesis, but the role of TRIM29 in cervical cancer is unclear. In this study, we first examined TRIM29 expression and found that TRIM29 mRNA and protein expression was upregulated in cervical cancer tissues when compared with the matched adjacent cervical tissues. We further detected TRIM29 protein with immunohistochemistry in 150 paraffin-embedded samples from early-stage cervical cancer patients. The results showed that high expression of TRIM29 was significantly associated with pelvic lymph node metastasis (*p*=0.002), advanced FIGO stage (*p*=0.026) and post-operative recurrence (*p*<0.001). Patients with high expression of TRIM29 had a shorter overall survival (HR 5.042, *p*<0.001) and disease-free survival (HR 4.260, *p*<0.001). TRIM29 was proven to be an independent prognostic factor for cervical cancer patients. When endogenous TRIM29 expression was knocked down by siRNAs, cell proliferation, colony formation, migration and invasion in cervical cancer cell lines HeLa and SiHa were obviously inhibited. Meanwhile, TRIM29 knockdown increased E-cadherin expression but decreased the expression of N-cadherin and β-Catenin, which indicated that TRIM29 could promote epithelial-mesenchymal transition (EMT). Mechanically, knockdown of TRIM29 enhanced GSK-3β protein expression and inhibited the expression of β-Catenin and C-myc proteins. GSK-3β is a key upstream suppressor of β-Catenin and c-myc expression is an indicator of Wnt/β-Catenin activity. Therefore, these results demonstrate that TRIM29 promotes tumor progression by activating Wnt/β-Catenin signaling. In conclusion, TRIM29 is overexpressed and associated with survival of early-stage cervical cancer, indicating that TRIM29 may be a potential prognostic biomarker and therapeutic target for cervical cancer.

## INTRODUCTION

Cervical cancer is the fourth most prevalent cancer in women worldwide, and in 2012, there was an estimated 527,600 new cases and 265,700 deaths from cervical cancer in the world [1], suggesting that it is a serious health problem for women. In order to enhance survival rate of patients with cervical cancer, identification of new biomarker for early diagnosis, evaluation of prognosis and personalized therapy is critical. For example, the International Federation of Gynaecology and Obstetrics (FIGO) staging system does not contain information about lymph node metastasis (LNM) [2] because the current imaging technique (such as MRI, CT or PET-CT) has low sensitivity and high negative likelihood ratio for detecting LNM [3–6]. However, LNM is an important prognostic factor in cervical cancer patients [7]. Therefore, there is an urgent need to identify biomarker for predicting LNM.

The tripartite motif (TRIM) protein family is characterized by containing evolutionary conserved domain structures, such as the RING finger, B-box and coiled-coil (RBCC) domain motifs [8]. In recent years, many members of this protein family were proved to play important roles in various biological processes including cell proliferation, differentiation, apoptosis, migration and oncogenesis [9–12].

TRIM29, a member of TRIM protein family, functions as a transcriptional regulator. Studies show that TRIM29 has been involved in a variety of cancers. Clinically, upregulated TRIM29 leads to tumor progression or poor prognosis in gastric cancer [13], prostate cancer [14], pancreatic adenocarcinoma [15], lung cancer [16, 17], nasopharyngeal carcinoma [18], as well as bladder cancer [19]. Mechanistically, we recently reported that TRIM29 expression promoted proliferation, EMT and metastasis of NPC cells via activating PTEN/AKT/mTOR signaling pathway [18]. In pancreatic adenocarcinoma cells, phosphorylated TRIM29 at Ser550 by MAPKAP kinase 2 induces radioresistance of cancer cells [15]. TRIM29 can enhance expression of DNMT3A by suppressing miR-29 in bladder cancer cells, leading to DNA methylation and silencing of the tumor suppressor PTEN [19]. The evidence suggests that TRIM29 functions as an oncogene. However, a few studies revealed that TRIM29 acts as a tumor suppressor in breast cancer cell lines by inhibiting TWIST1 and epithelial-mesenchymal transition (EMT) [20, 21]. Therefore, TRIM29 may have the different roles in different types of cancer.

Recently, TRIM29 has been proved to decrease adhesion and promote invasion of cervical cancer cells through activating p63-mediated pathway [22]. However, the clinical significance of TRIM29 in cervical cancer is not clear and its biological role in cervical cancer remains to be further elucidated. In this study, we focused on investigation of TRIM29 expression and its clinical significance and biological function in cervical cancer and also explored which cell signaling pathway was regulated by TRIM29 in cervical cancer cells.

## RESULTS

### TRIM29 is overexpressed in cervical cancer tissues

**Figure 1:**
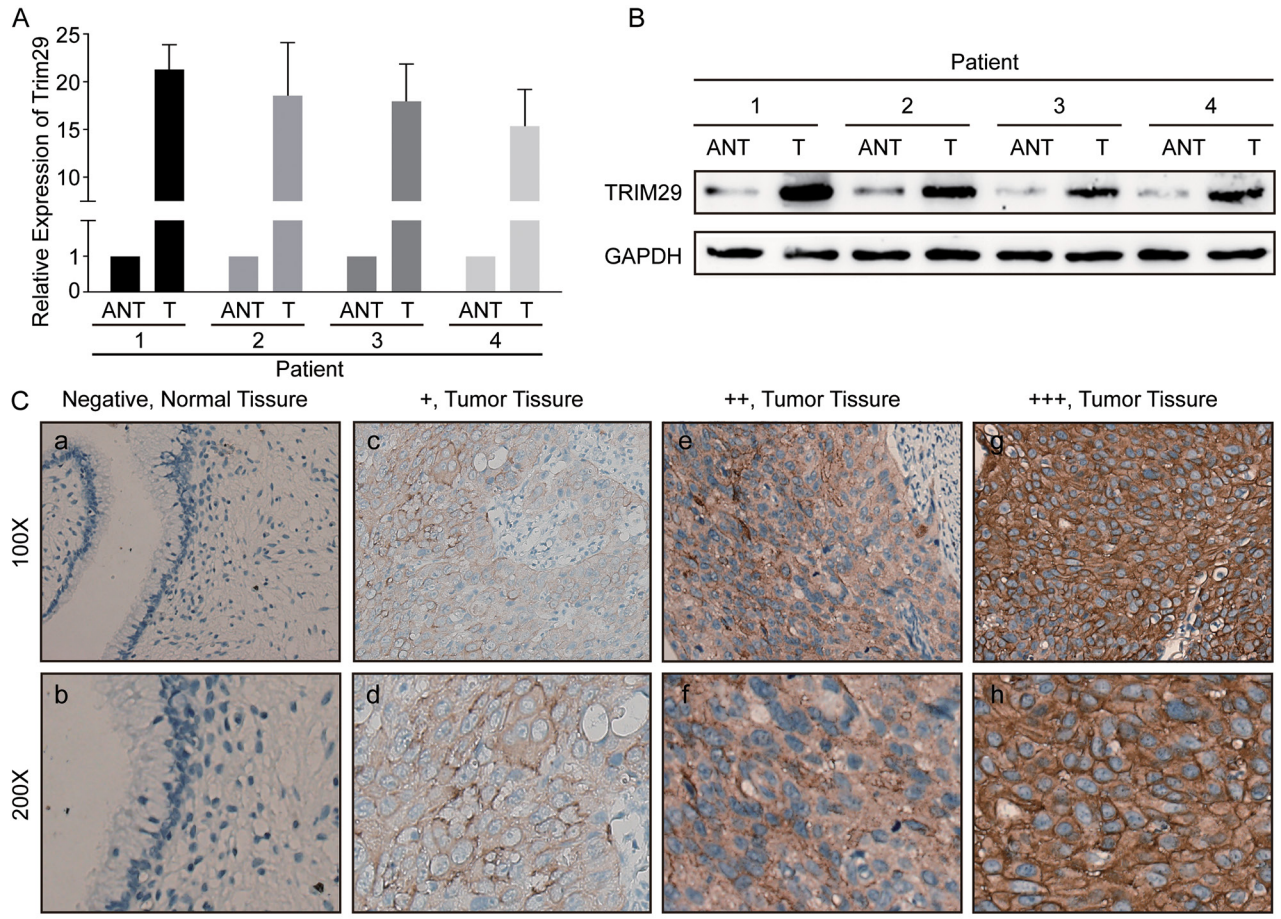
TRIM29 is overexpressed in cervical cancer tissues **A.** Relative expression of TRIM29 mRNA in cervical tissues (T) and paired adjacent normal tissues (ANT). TRIM29 mRNA expression was examined by qPCR and normalized to GAPDH expression. **B.** The image of western blotting analyses of TRIM29 protein expression in four pairs of cervical cancer tissues (T) and adjacent normal tissues (ANT). GAPDH was used as the loading control. **C.** Immunohistochemical staining of TRIM29 protein in normal cervical tissues and cervical cancer tissues with different staining intensities.

To investigate whether TRIM29 is dysregulated in human cervical cancer, we performed qRT-PCR and western blotting on four pairs of fresh cervical cancers (T) and matched adjacent cervical tissues (N). As showed in Figure 1A, TRIM29 mRNA expression was increased by 15.88 to 22.82 folds in the four cervical cancer tissues compared with the four matched adjacent cervical tissues. As expected, the TRIM29 protein expression was also markedly up-regulated in the four cervical cancer tissues, as judged by western blot (Figure 1B).

### TRIM29 protein expression is associated with malignant phenotypes and poor survival of cervical cancer patients

To explore the clinical significance of TRIM29 overexpression, immunohistochemical staining (IHC) was used to detect TRIM29 protein expression in 150 paraffin-embedded cervical cancer samples. In the cervical cancer tissues, TRIM29 protein was primarily located in the cytoplasm and membrane of cervical cancer cells. However, no staining was observed in the normal tissues (Figure 1C).

According to the IHC score (see Material and Methods) of each patients, 62 (41.3%) patients were assigned to the high-expression group and 88 (58.7%) patients to the low-expression group. Correlation analysis showed that TRIM29 protein overexpression was significantly associated with advanced FIGO Stage (*p*=0.026), pelvic lymph node metastasis (PLNM, *p*=0.002) and cancer relapse (*p*<0.001) (Table 1). No correlation was found between TRIM29 expression and other clinicopathological features.

**Table 1:** Correlation between TRIM29 expression and clinicopathological characteristics in patients with early-stage cervical cancer

Clinicopathological characteristics	N (%)	TRIM29 Expression		*P* value*
		Low expression	High expression	
Age (years)				
≤46	70 (46.7)	42	28	0.868
>46	80 (53.3)	46	34	
Squamous cell carcinoma antigen (ng/ml)				
≤1.5	90 (60.0)	56	34	0.312
>1.5	60 (40.0)	32	28	
FIGO staging system				
Stage Ia	13 (8.7)	8	5	0.026
Stage Ib	92 (61.3)	61	31	
Stage IIa	45 (30.0)	19	26	
Types of tumor growth				
Exogenous	80 (53.3)	49	31	0.711
Endogenous	36 (24.0)	21	15	
Ulcerative	34 (22.7)	18	16	
Histological types				
Squamous cell carcinoma	145 (96.7)	84	61	0.555
Adenocarcinoma	4 (2.7)	3	1	
Adenosquamous carcinoma	1 (0.6)	1	0	
Pathological grade				
G1	20 (13.3)	14	6	0.384
G2	117 (78.0)	68	49	
G3	13 (8.7)	6	7	
Carcinoma diameter (cm)				
≤4	113 (75.3)	69	44	0.399
>4	37 (24.7)	19	18	
Myometrium invasion				
<1/2	61 (40.7)	32	29	0.238
≥1/2	89 (59.3)	56	33	
Infiltration of parauterine organ				
No	144 (96.0)	86	58	0.231
Yes	6 (4.0)	2	4	
Property of surgical margin				
Negative	137 (91.3)	81	56	0.773
Positive	13 (8.7)	7	6	
Lymphovascular space involvement				
No	127 (84.7)	76	51	0.500
Yes	23 (15.3)	12	11	
Pelvic lymph node metastases				
No	108 (72.0)	72	36	0.002
Yes	42 (28.0)	16	26	
Relapse				
No	111 (74.0)	76	35	<0.001
Yes	39 (26.0)	12	27	
Adjuvant Therapy				
No	48 (32.0)	30	18	0.513
Yes	102 (68.0)	58	44	

* *P* values are calculated by χ2 test or Fisher's exact test.

**Figure 2:**
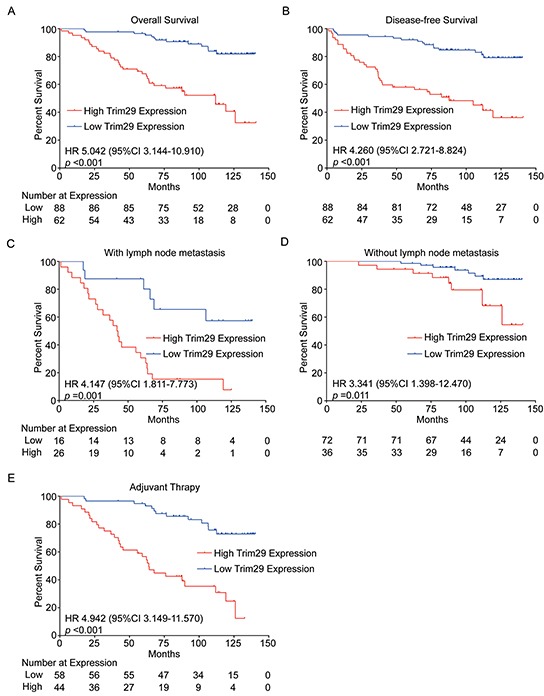
TRIM29 expression is associated with survival of cervical cancer patients **A.** and **B.** Kaplan-Meier survival analysis of 150 patients with high or low TRIM29 expression. **C.** and **D.** Kaplan-Meier survival analysis of patients with high or low TRIM29 expression in subgroups with or without lymph node metastasis. **E.** Kaplan-Meier survival analysis of patients with high and low TRIM29 expressions in subgroup received adjuvant therapy after surgery.

Survival analysis indicated that patients with high TRIM29 expression had a significantly shorter overall survival (OS) (HR 5.042, 95% CI 3.144-10.910, *p*<0.001) or disease free survival (DFS) (HR 4.260, 95% CI 2.721-8.824, *p*<0.001) than those with low expression level (Figure 2A, 2B).

PLNM plays a very important role in the prognosis of cervical cancer patients. Survival analysis in the subgroup with PLNM showed that patients with high TRIM29 expression suffered a shorter OS (HR 4.147, 95% CI 1.811-7.773, *p*=0.001, Figure 2C). Meanwhile, a similar result was observed in the subgroup without PLNM (HR 3.341, 95% CI 1.398-12.470, *p*=0.011, Figure 2D). This suggests that higher expression of TRIM29 is always associated with poor survival in cervical cancer patients, no matter whether or not they have PLNM.

In clinical practice, radical hysterectomy (hysterectomy plus pelvic lymph node dissection) followed or not followed by adjuvant chemoradiotherapy is a standard treatment for early-stage cervical cancer patients [23, 24]. In our study, 102 patients received adjuvant chemoradiotherapy and 48 patients did not. We found that higher TRIM29 expression was significantly correlated with a shorter OS (HR 4.942, 95% CI 3.149-11.570, *p*<0.001) in patients with adjuvant therapy (Figure 2E) but not in those without adjuvant therapy (data not shown), the difference might be caused by different sample sizes of the two subgroups.

Furthermore, univariate analysis revealed that high TRIM29 expression, advanced FIGO Stage, and PLNM were significant predictors for poor OS (Table 2) and DFS (Table 3) in cervical cancer patients. Multivariate analysis indicated that TRIM29 expression, FIGO Stage and PLNM were also independent prognostic factors for OS (Table 2) and DFS (Table 3) in the patients.

**Table 2:** Univariate and multivariate analysis of the effects of TRIM29 expression and clinicopathological characteristics on overall survival in patients with early-stage cervical cancer

Clinicopathologic features	Univariate cox analysis		Multivariate cox analysis	
	HR (95% CI)	*P*	HR (95% CI)	*P*
Age (>46 years vs. ≤46 years)	1.679 (0.844-3.337)	0.140		
SCC-Ag(>1.5ng/ml vs. ≤1.5ng/ml)	0.863 (0.428-1.740)	0.681		
Pathological grade (I, II, III)	0.820 (0.381-1.764)	0.612		
Carcinoma diameter (>4cm vs. ≤4cm)	1.143 (0.542-2.410)	0.725		
Myometrium invasion (Yes vs. No)	1.336 (0.444-4.024)	0.606		
IPO (Yes vs. No)	1.296 (0.098-17.140)	0.844		
Positive surgical margin (Yes vs. No)	0.245 (0.029-2.075)	0.197		
LVSI (Yes vs. No)	0.860 (0.394-1.878)	0.704		
PLNM (Yes vs. No)	5.447 (2.536-11.701)	<0.001	5.076 (2.572-10.020)	<0.001
FIGO staging system (IIa, Ib, Ia)	7.740 (3.456-17.336)	<0.001	6.721 (3.290-13.729)	<0.001
TRIM29 Expression (High vs. Low)	3.237 (1.551-6.755)	0.002	3.076 (1.544-6.129)	0.001

Abbreviations: SCC-Ag, Squamous cell carcinoma antigen; IPO, Infiltration of parauterine organ; LVSI, Lymphovascular space involvement; PLNM, Pelvic lymph node metastases.

**Table 3:** Univariate and multivariate analysis of effects of TRIM29 expression and clinicopathological features on disease-free survival in patients with early-stage cervical cancer

Clinicopathologic features	Univariate cox analysis		Multivariate cox analysis	
	HR (95% CI)	*P* value	HR (95% CI)	*P* value
Age (>46 years vs. ≤46 years)	1.643 (0.865-3.120)	0.130		
SCC-Ag(>1.5ng/ml vs. ≤1.5ng/ml)	0.822 (0.420-1.606)	0.566		
Pathological grade (I, II, III)	0.904 (0.444-1.841)	0.780		
Carcinoma diameter (>4cm vs. ≤4cm)	1.198 (0.604-2.376)	0.605		
Myometrium invasion (Yes vs. No)	1.327 (0.494-3.562)	0.575		
IPO (Yes vs. No)	1.296 (0.098-17.140)	0.782		
Positive surgical margin (Yes vs. No)	0.613 (0.127-2.954)	0.542		
LVSI (Yes vs. No)	1.029 (0.494-2.147)	0.938		
PLNM (Yes vs. No)	3.091 (1.493-6.400)	0.002	2.877 (1.525-5.428)	0.001
FIGO staging system (IIa, Ib, Ia)	7.720 (3.679-16.201)	<0.001	7.366 (3.836-14.145)	<0.001
TRIM29 Expression (High vs. Low)	2.923 (1.455-5.872)	0.003	2.830 (1.495-5.356)	0.001

Abbreviations: SCC-Ag, Squamous cell carcinoma antigen; IPO, Infiltration of parauterine organ; LVSI, Lymphovascular space involvement; PLNM, Pelvic lymph node metastases.

### TRIM29 knockdown inhibits proliferation and colony formation of cervical cancer cells

**Figure 3:**
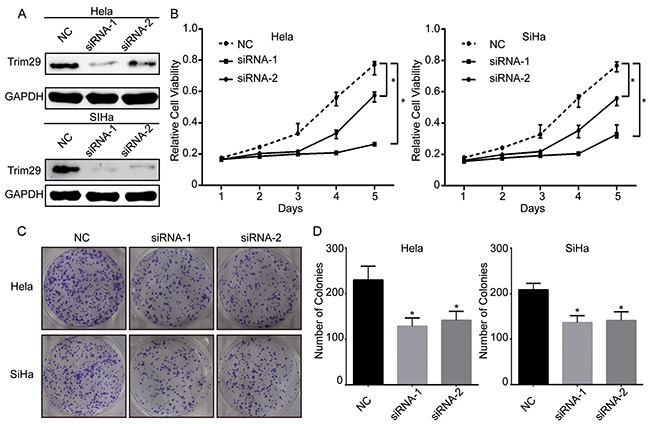
Knockdown of TRIM29 expression suppresses the viability of cervical cancer cells **A.** The protein level of TRIM29 in Hela and SiHa cells after transfection of siRNAs against TRIM29 or scramble sequence was analyzed by Western blot. GAPDH was used as the loading control. **B.** Growth curves of Hela and SiHa cells with transfected siRNAs or scramble sequence. Cell growth was determined by MTT. **C.** and **D.** A colony-formation assay was performed to determine the oncogenic growth of Hela and SiHa cells transfected with siRNAs or scramble sequence. *p<0.05.

The above results demonstrate that overexpressed TRIM29 is correlated with tumor progression and poor survival of cervical cancer. Thus, we want to explore whether knockdown of TRIM29 can inhibit growth of cervical cancer cells. Two siRNAs were designed against TRIM29 and transfected into cervical cancer cell lines Hela and SiHa. Western blot verified that TRIM29 protein was downregulated in both HeLa and SiHa cells transfected with the two siRNAs (Figure 3A). Cell proliferation assay showed that proliferation of both HeLa and SiHa cells transfected with siRNA-1 or -2 was remarkably inhibited compared with that of those transfected with control siRNA (Figure 3B), indicating that TRIM29 expression could promote cancer cell proliferation while TRIM29 downregulation suppressed cell growth. To confirm the effect of TRIM29 knockdown on the oncogenic growth of cervical cancer cells, plate colony formation assay was also conducted on the two cells. As expected, the colony numbers of the both cells with TRIM29 knockdown were significantly less than those of the negative control cells (Figure 3C, 3D). These findings suggest that knockdown of TRIM29 represses proliferation and survival ability of cervical cancer cells.

### Downregulation of TRIM29 suppresses migration and invasion of cervical cancer cells

**Figure 4:**
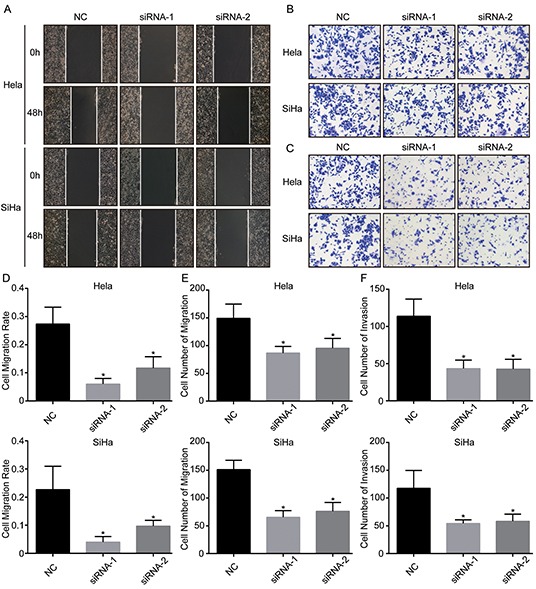
TRIM29 knockdown inhibits migration and invasion of cervical cancer cells **A.** and **D.** Wound healing assays were performed to determine the migratory abilities of si-TRIM29-transfected Hela and SiHa cells. **B.** and **E.** Transwell assays were performed to determine the migratory abilities of si-TRIM29-transfected Hela and SiHa cells. **C.** and **F.** Transwell assays with matrigel were performed to determine the invasive abilities of si-TRIM29-transfected Hela and SiHa cells. *p<0.05.

The above clinical analysis showed that TRIM29 overexpression was associated with PLNM and short DFS, which suggested that TRIM29 could promote tumor progression and metastasis of cervical cancer. It is well known that cancer cells can metastasize only when they possess the ability of migration and invasion. Therefore, we carried out in vitro experiments to test whether TRIM29 enhances migration and invasion of cervical cancer cells. Wound healing assay showed that the decreased expression of TRIM29 by the two siRNAs led to slower migration rate in both cervical cancer cell lines (Figure 4A, 4D). Transwell migration assay also displayed that the migrated cells were much less in TRIM29 knockdown cells than in control cells (Figure 4B, 4E). More important, transwell invasion assay indicated that inhibition of TRIM29 could significantly reduce the number of invaded cells in both cervical cancer cells (Figure 4C, 4F). These results suggest that TRIM29 promotes migration and invasion of cervical cancer cells, while knockdown of TRIM29 inhibits migration and invasion, which lead to metastasis.

### TRIM29 knockdown decreases epithelial-mesenchymal transition by inhibiting Wnt/β-Catenin pathway

**Figure 5:**
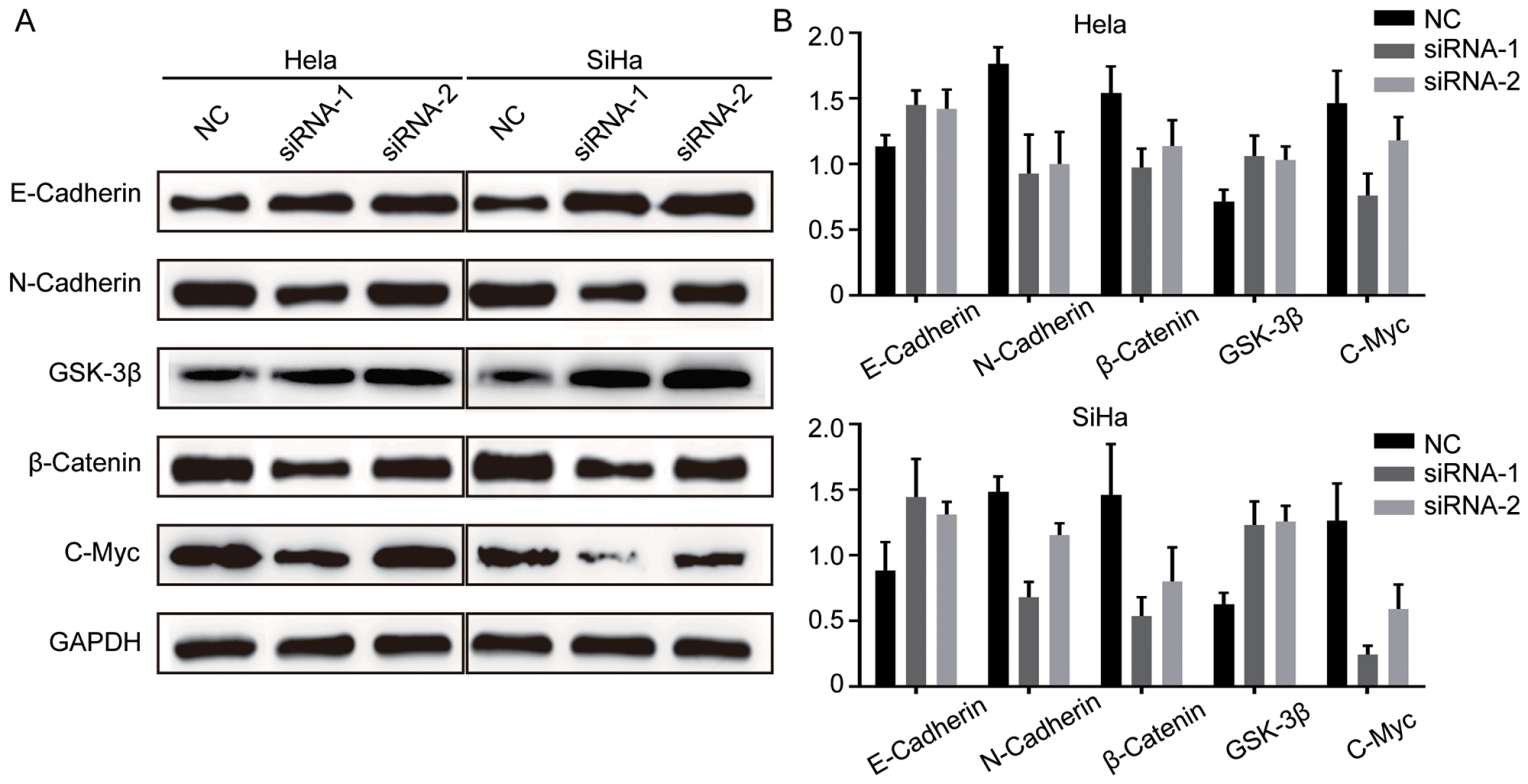
TRIM29 knockdown inhibits EMT and inactivates Wnt/β-Catenin pathway in cervical cancer cells **A.** and **B.** Analysis of E-cadherin, N-cadherin, β-catenin, GSK-3β and C-myc expression by western blot in Hela and SiHa cells treated with siRNA-TRIM29.

Studies demonstrate that before metastasis, cancer cells may have epithelial-mesenchymal transition (EMT), by which cancer cells lose cell-cell adhesion, apical-basolateral polarity and epithelial markers, and acquire motility, spindle-cell shape and mesenchymal markers [25]. Thus EMT is thought to facilitate cancer cell motility, invasion and metastasis. Based on this knowledge and our results above, we hypothesize that TRIM29 can induce EMT during its promotion of migration and invasion. To this end, we first explored the markers of EMT with Western blot: E-cadherin (epithelial marker), N-cadherin and β-catenin (mesenchymal markers). The result showed that TRIM29 knockdown upregulated E-cadherin and repressed N-cadherin and β-catenin (Figure 5), which clearly indicates inhibition of EMT in both cervical cancer cells. Recently, increasing evidence shows that Wnt/β-catenin pathway is involved in cancer metastasis. β-catenin has been reported to be aberrantly accumulated in human tumors, while GSK-3β can induce ubiquitination and proteasomal degradation of β-catenin [26, 27]. In addition, c-myc expression is thought to be an indicator of Wnt/β-Catenin activity. Therefore, we simultaneously examined the expression of GSK-3β, c-myc and β-catenin when TRIM29 was knocked down. Not unexpectedly, the GSK-3β expression was obviously elevated while c-myc and β-catenin expression was also markedly reduced when TRIM29 was knocked down (Figure 5), which indicates that the activity of Wnt/β-catenin pathway was inhibited. These results suggest that TRIM29 may promote EMT, migration, invasion and metastasis of cervical cancer via activating Wnt/β-Catenin signaling.

## DISCUSSION

In recently years, several members of TRIM family had been characterized to be function as oncogenes or tumor suppressors. TRIM29, as mentioned above, mainly acts as an oncogene in most tumors and also may function as a tumor suppressor in few cancers, which should be further confirmed.

In this study, we demonstrate that TRIM29 is overexpressed in cervical cancer and correlated with PLNM, advanced FIGO stage, postoperative recurrence and poor survival of patients, suggesting TRIM29 plays an oncogenic role in cervical cancer. This result is consistent with other reports on TRIM29 in most cancers. Importantly, TRIM29 expression was proven to be an independent prognostic factor for poor OS and DFS in cervical cancer patients, indicating that TRIM29 is a potential biomarker for prognostic prediction in cervical cancer, which may help identify a more aggressive subtype of patients that need to accept postoperative adjuvant chemoradiotherapy. Furthermore, as we know, early FIGO stage patients with PLNM have a poor prognosis [28–30], and we find that 41.9% of patients with high TRIM29 expression have PLNM, while only 18.8% of patients with low TRIM29 expression have PLNM. Therefore, TRIM29 may be a potential biomarker for evaluation of PLNM in cervical cancer patients. In addition, in the patients who need post-operative adjuvant chemoradiotherapy, those with high TRIM29 expression show a significantly shorter OS than those with low TRIM29 expression, indicating that those with high TRIM29 expression require more aggressive or different adjuvant chemoradiotherapy regimen. This suggests that TRIM29 also may be potential biomarker for personalized therapy in cervical cancer.

Although some studies on TRIM29 in cancer have been reported, little is known about the biological function and mechanism of TRIM29 in cancer. Until now, only Masuda Y group reported a study of TRIM29 in cervical cancer cells as aforementioned [22]. In this study, we reveal the extensive biological functions of TRIM29 in cervical cancer cells, such as promotion of proliferation, colony formation, migration, invasion and EMT of cervical cancer cells. Of those functions, promotion of cancer cell invasion by TRIM29 is in accordance with that reported by Masuda Y. More important, we elucidate that TRIM29 downregulation suppresses EMT, migration and invasion of cervical cancer cells by inhibiting Wnt/β-Catenin pathway. These results will help us understand how TRIM29 expression leads to tumor progression, PLNM and poor prognosis in clinical patients with cervical cancer and provide a potential molecular target for cervical cancer therapy.

In conclusion, our results, demonstrated for the first time that TRIM29 is overexpressed in cervical cancer and is a potential prognostic biomarker of cervical cancer, which provides additional information for guiding therapeutic strategy. Our results warrant further studies on the detailed mechanisms by which TRIM29 promotes tumor progression in cervical cancer.

## MATERIALS AND METHODS

### Cell culture and antibodies

Two cervical cell lines (HeLa and SiHa) were maintained in our laboratory. The cells were cultured in DMEM (Gibco) medium with 10% FBS (Gibco) at 37°C in an atmosphere of 5% CO_2_. Rabbit polyclonal antibodies to TRIM29 and C-Myc were purchased from Abcam (USA). Purified Mouse Anti-E-Cadherin, N-cadherin and β-catenin were purchased from BD Biosciences (USA). Anti-rabbit and anti-mouse HRP-conjugated secondary antibodies were obtained from Cell Signaling Technology (USA).

### Samples and clinical characteristics

This study was performed on a total of 150 paraffin-embedded cervical cancer samples collected from the Department of Pathology of Sun Yat-Sen University Cancer Center, Guangzhou, China. Four pairs of fresh tumor tissues and matched normal cervical tissues were obtained from Gynecology and Obstetrics Department, Women and Children's Medical Center, Guangzhou in 2015. In this study, all 150 patients were first diagnosed in this cancer center between 2005 and 2010. The clinical staging and pathological classification were determined according to the 2009 FIGO criteria. Patients enrolled in this study were pathologically diagnosed as squamous cell carcinoma, adenocarcinoma or both, and not treated with radiotherapy, chemotherapy, or hormonal therapy before surgery. Clinical characteristics of all patients are summarized in Table 1. The follow-up time for all patients with cervical cancer ranged from 1 to 141 months. Overall survival (OS) is defined as the time from surgery until death due to any cause or the last follow-up. Disease-free survival (DFS) is defined as the time from the surgery to the first relapse or metastasis or death due to any cause or the last follow-up. This study was reviewed and approved by Sun Yat-Sen University Cancer Center Institutional Review Board. A written informed consent from every patient was obtained before surgery.

### Immunohistochemical staining

Paraffin-embedded tissue samples were cut into 4-μm thick sections, and these sections were deparaffinized with dimethylbenzene and rehydrated. The sections were boiled in 0.01M sodium citrate-hydrochloric acid buffer solution (pH = 6.0) for 15 mins in a microwave oven to retrieve antigen. Then the sections were treated with 3% hydrogen peroxide to inactive endogenous peroxidase. Then, these sections were incubated with an anti-TRIM29 rabbit polyclonal antibody (1:100) overnight at 4°C. After washing with PBST, the sections were then incubated with an anti-rabbit HRP-conjugated secondary antibody at room temperature for 30 mins. Finally, all the sections were developed in 3-amino-9-ethylcarbazole for 15 min and counterstained with hematoxylin. Sections incubated with normal goat serum overnight at 4°C were used as negative control.

The staining intensity of immunostained sections was evaluated independently by two pathologists who were blinded to the patients' clinical information. The TRIM29 immunostaining score was based on the product of the percentage of positive tumor cells multiplied by the intensity of staining. The percentage of positive tumor cells was scored as 1 (<10%), 2 (10-50%), 3 (50-75%), and 4 (>75%). The intensity of staining was graded as 0 (no staining), 1(weak), 2 (moderate), or 3 (strong). Therefore, the final immunostaining score for every patient was ranged from 0 to 12. Expression levels of TRIM29 were defined as follows: low expression, final score 0 – 3; high expression, final score 4-12. Based on the criterion, all patients were divided into two groups: low TRIM29 expression group (final score 2 – 3) and high TRIM29 expression group (final score 4 – 12).

### Knockdown of TRIM29

Small interfering RNAs (siRNAs) against TRIM29 were designed and purchased commercially (Genepharma, China) as follows: TRIM29 siRNA-1, 5′-GAGCUGCGCAAGUCCAUUUTT-3′; TRIM29 siRNA-2, 5′-ACGGAGCUGUCAUUGCAAATT-3′; Scramble siRNA, UUCUCCGAACGUGUCACGUTT. These siRNAs were transfected into HeLa and SiHa cells using Lipofectamine 2000 (Invitrogen) in the absence of serum according to the manufacturer's instruction, when cells were reaching 40%∼60% confluence. After 48 h, this medium was replaced with DMEM and cells were cultured for another 24h. Then the cells were harvested to analyze the efficiency of TRIM29 knockdown.

### Western blot

Proteins (30 μg) extracted from cervical cancer cells and tissues were loaded and separated on 10% SDS-PAGE. After electrophoresis, proteins were transferred onto Immobilon-P Transfer Membrane (Millipore, Billerica, MA). Then the membranes were blocked with 5% non-fat milk in TBST (with 0.1% Tween-20) and incubated with the indicated primary antibodies overnight at 4°C. After washing with TBST, the membranes were then incubated with secondary antibodies at room temperature for 60 mins. Finally the signals were detected by Western Lightning Plus-ECL Kit (PerkinElmer, MA, USA) according to manufacturer's instructions.

### RNA isolation and real-time quantitative RT-PCR analysis

Total RNA was isolated from cervical cancer cells using Trizol (Invitrogen) according to the manufacturer's protocol. Two micrograms of total RNA was subjected to a reverse transcription (RT) system (Promega, USA) for first-strand cDNA synthesis in a final volume of 20 μl. Then 2 μl of cDNA was used to perform quantitative PCR reaction with GoTaq qPCR Master Mix (Promega, USA) on Roche LightCycle96. GAPDH was used as the normalization control and all qRT-PCR reactions were done in triplicate. The following primer sequences were used: TRIM29 forward, 5′-TGCGAGCTGCATCTCAAGC-3′; TRIM29 reverse, 5′-GGTGCTATGATTCTTGTGCTCC-3′; GAPDH forward, 5′-GGAGCGAGATCCCTCCAAAAT-3′; GAPDH forward, 5′-GGCTGTTGTCATACTTCTCATGG-3′. 2^−ΔΔCt^ was used to represent expression changes.

### Cell proliferation assay

Cells were plated onto 96-well plates at 1000 cells per well and incubated for 1-5 days. During the incubation, 10 μl MTT Reagent was added to the wells that would be measured every day and cultured for another 4 hours. Then the supernatant was discarded and 200 μl of DMSO was added to each well. Finally the absorbance of the solution was measured at 570 nm in a multifunctional microplate reader. The growth curve was constructed with time (day) as abscissa and absorbance as ordinate.

### Plate colony formation assay

Cells treated by TRIM29-siRNAs or scramble control were plated onto 6-well plates (1000 cells/well). The cells were then cultured at 37°C and 5% CO_2_ for 2 weeks until visible clones were formed. The medium was changed every three days during this time. The clones were washed with PBS for three times and fixed with 4% paraformaldehyde for 15 min. Then the clones were stained with 0.1% crystal violet solution for 30 min. After washing, the number of colonies was counted under a microscope.

### Wound healing assay

Cells treated by TRIM29-siRNAs or scramble control were seeded in 6-well plates (3 × 10^5^ cells/well) and cultured for 24 hours until 90%-100% confluence. Then a pipette tip was used to make a straight scratch and the images were acquired. At 48 hour after incubating in serum-free medium, the cultured cells were imaged again. Cell migration rates were determined from all the images acquired above.

### Transwell migration and invasion assays

Cell migration and invasion assays were performed on transwell chamber with 8-μm pore-size filters without (for migration) or with (for invasion) coated matrigel (Falcon). Cells were trypsinized and resuspended in serum-free medium. 250 μl of cell suspension (1 × 10^5^cells) was added to the upper chamber in a transwell insert and 750 μl medium with 10% FBS was added to the lower chamber in a 24-well plate. Then the transwell inserts were placed into the wells in the 24-well plate and cultured at 5% CO_2_ at 37°C for 16 hours. After that, the cells on the top of the membrane were removed with a cotton swabs, and the attached cells on the underside of the membrane were stained with 0.1% crystal violet. After washing clones with PBS, the number of cells was calculated under the microscope.

### Statistical analysis

Kaplan-Meier method was used to analyze overall survival and disease-free survival with GraphPad Prism 6. Chi-square test was applied to analyze the correlation between TRIM29 expression and the clinicopathological features. Two-tailed, unpaired Student's t-test was used to compare the differences between experimental and control groups in vitro assays. Univariate and multivariate analysis (backward selection) were used to analyze the correlation of TRIM29 expression and clinicopathological features. Results are shown as mean values with 95% confidence intervals. Error bars represents standard deviation. A value of *p* <0.05 was considered significant. All other statistical analyses were conducted using the SPSS19.0.
